# Supplementary material for: Adipocyte p53 coordinates the response to intermittent fasting by regulating adipose tissue immune cell landscape
Source: Nat Commun. 2024 Feb 15;15:1391. doi: 10.1038/s41467-024-45724-y (PMC10869344; doi:10.1038/s41467-024-45724-y)
Supplement: Supplementary file 1 — Supplementary Information [file 41467_2024_45724_MOESM1_ESM.pdf]

Supplementary information

Adipocyte p53 coordinates the response to intermittent fasting by regulating adipose tissue immune cell landscape

Isabel Reinisch[1,2], Helene Michenthaler[1], Alba Sulaj[3,4], Elisabeth Moyschewitz[1], Jelena Krstic[1], Markus Galhuber[1], Ruonan Xu[1], Zina Riahi[1], Tongtong Wang[2], Nemanja Vujic[5], Melina Amor[5], Riccardo Zenezini Chiozzi[6], Martin Wabitsch[7], Dagmar Kolb[1,8], Anastasia Georgiadi[3], Lisa Glawitsch[9], Ellen Heitzer[9], Tim J. Schulz[10, 11, 12], Michael Schupp[13], Wenfei Sun[14], Hua Dong[15], Adhideb Ghosh[2,16], Anne Hoffmann[17], Dagmar Kratky[5,18], Laura C Hinte[19], Ferdinand von Meyenn[19], Albert J.R. Heck[6], Matthias Blüher[20], Stephan Herzig[3,4], Christian Wolfrum[2], Andreas Prokesch[1,18,*]

[1] Gottfried Schatz Research Center for Cell Signaling, Metabolism and Aging, Division of Cell Biology, Histology and Embryology, Medical University of Graz, Graz, Austria.
[2] Institute of Food Nutrition and Health, Department of Health Sciences and Technology, Eidgenössische Technische Hochschule Zürich (ETH), Schwerzenbach, Switzerland.
[3] Institute for Diabetes and Cancer, Helmholtz Munich, German Center for Diabetes Research (DZD), Neuherberg, Germany.
[4] Department of Endocrinology, Diabetology, Metabolism and Clinical Chemistry (Internal Medicine 1), Heidelberg University Hospital, Heidelberg, Germany.
[5] Gottfried Schatz Research Center, Molecular Biology and Biochemistry, Medical University of Graz, Graz, Austria.
[6] Biomolecular Mass Spectrometry and Proteomics, Bijvoet Center for Biomolecular Research and Utrecht Institute of Pharmaceutical Sciences, Utrecht University, Utrecht, Netherlands.
[7] Division of Pediatric Endocrinology and Diabetes, Department of Pediatrics and Adolescent Medicine, University Medical Center Ulm, Ulm, Germany.
[8] Core Facility Ultrastructure Analysis, Medical University of Graz, Graz, Austria.
[9] Institute of Human Genetics, Diagnostic and Research Center for Molecular BioMedicine, Medical University of Graz, Graz, Austria.
[10] Department of Adipocyte Development and Nutrition, German Institute of Human Nutrition, Nuthetal, Germany.
[11] German Center for Diabetes Research (DZD), München-Neuherberg, Germany.
[12] University of Potsdam, Institute of Nutritional Science, Nuthetal, Germany.
[13] Institute of Pharmacology, Max Rubner Center (MRC) for Cardiovascular Metabolic Renal Research, Charité-Universitätsmedizin Berlin, Corporate Member of Freie Universität Berlin, Humboldt-Universität zu Berlin, Berlin, Germany.
[14] Department of Bioengineering, Stanford University, CA, USA.
[15] Stem cell Bio Regenerative Med Institute, University of Stanford, CA, USA.
[16] Functional Genomics Center Zurich, Eidgenössische Technische Hochschule Zürich (ETH), Zurich, Switzerland.
[17] Helmholtz Institute for Metabolic Obesity and Vascular Research (HI-MAG) of the Helmholtz Center Munich at the University of Leipzig and University Hospital Leipzig, Leipzig, Germany.
[18] BioTechMed-Graz, Graz, Austria
[19] Laboratory of Nutrition and Metabolic Epigenetics, Institute for Food, Nutrition and Health, Department of Health Sciences and Technology, ETH Zurich, Zurich, Switzerland.
[20] Department of Medicine, University of Leipzig, Leipzig, Germany.
* Correspondence: andreas.prokesch@medunigraz.at

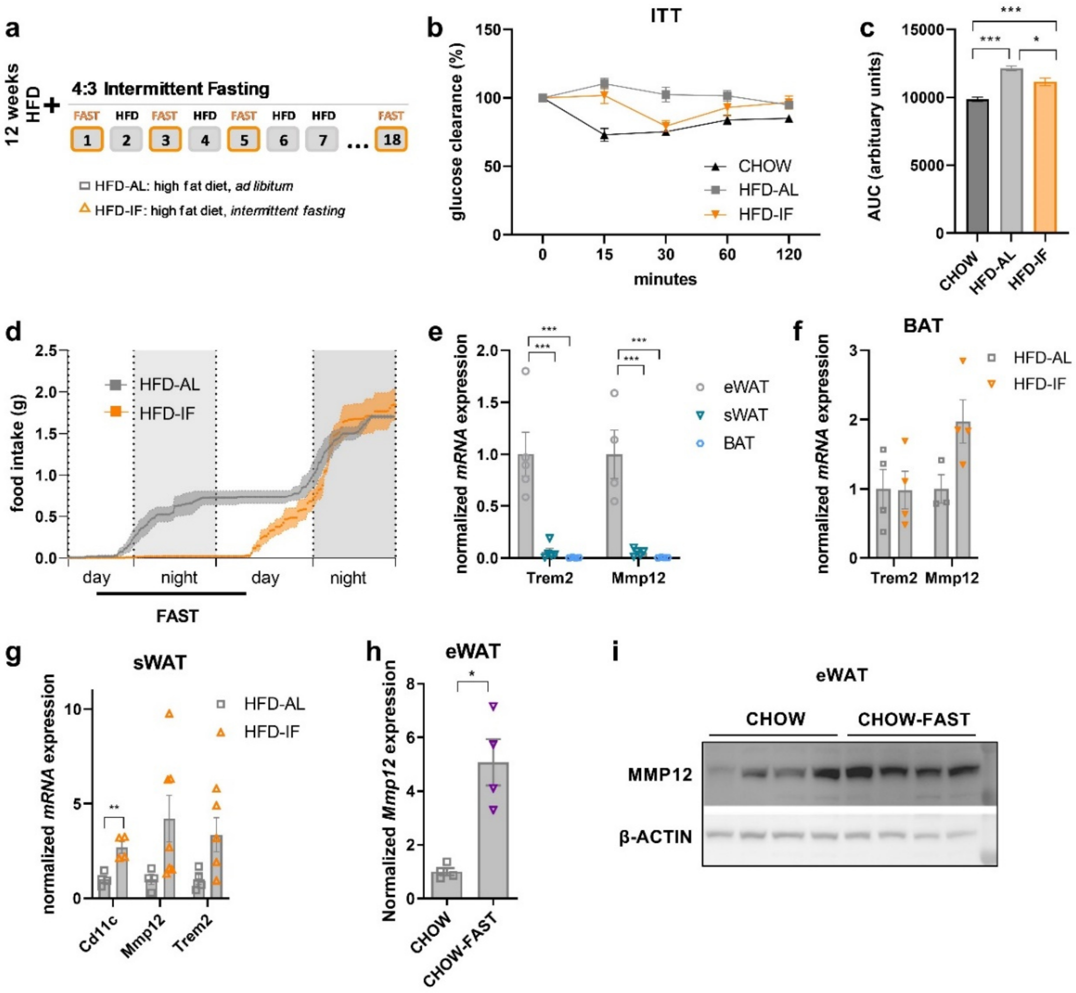

Supplementary Fig. 1 | Intermittent fasting increases the abundance of crown-like structures in AT. **a** Experimental design of 4:3 days/week intermittent fasting for 18 days in 12 weeks high-fat diet (HFD)-fed mice. **b, c** Insulin tolerance test (ITT) of lean (n = 8 mice), HFD-AL (n = 6 mice), and HFD-IF (n = 4 mice) mice. **d** Food intake measured in metabolic cages of HFD-AL and HFD-IF (n=3 mice per group) mice during a time span of 48 h. **e** mRNA expression levels of *Trem2* and *Mmp12* in eWAT (n=5 mice), sWAT (n=5 mice), and BAT (n=4 mice) of HFD-IF mice. **f** mRNA expression levels of *Trem2* and *Mmp12* in BAT of HFD-AL and HFD-IF mice (n=4 mice per group). **g** mRNA expression levels of *Cd11c*, *Mmp12* and *Trem2* in sWAT of HFD-AL (n=4 mice) and HFD-IF (n=4-7 mice) mice. **h** *Mmp12* mRNA expression levels in eWAT of lean, chow-fed (CHOW) or overnight fasted (CHOW-FAST) mice (n=4 mice per group). **i** Western blot analysis using anti-MMP12 and antibody (anti-β-actin as loading control) in eWAT of CHOW or CHOW-FAST mice. Data are presented as mean values ± SEM. Significant differences were analysed by two-tailed, unpaired t-test (**f, g, h**), or one-way (**c, e**) or two-way (**b**) ANOVA with Bonferroni post hoc tests. ***P < 0.001, **P < 0.01 and *P < 0.05. Source data are provided as a Source Data file.

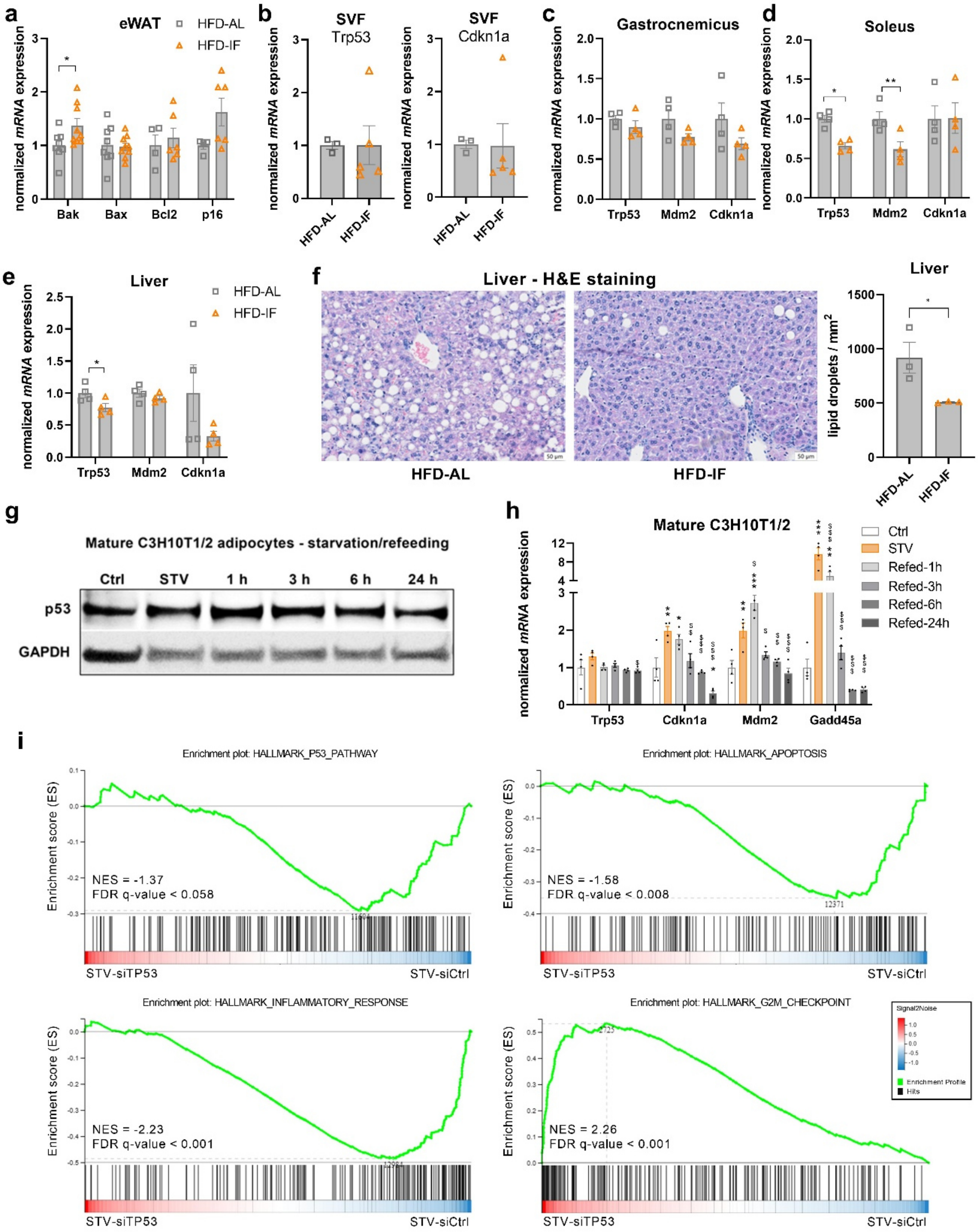

Supplementary Fig. 2 | IF elicits AT stress response via adipocyte-autonomous p53 signalling. a mRNA expression levels of genes encoding for apoptotic markers or senescence-associated genes in bulk eWAT of HFD-AL (n=8 mice for *Bak* and *Bax*; n=4 mice for *Bcl2* and *p16*) and HFD-IF (n=9 mice for *Bak* and *Bax*; n=6 mice for *Bcl2* and *p16*) mice. **b** mRNA expression levels of *Trp53* and *Cdkn1a* in the stromal vascular fraction (SVF) isolated from eWAT of HFD-AL (n=3 mice) and HFD-IF (n=5 mice) mice. **c-e** mRNA expression levels of *Trp53*, *Mdm2* and *Cdkn1a* in gastrocnemius (**c**), soleus (**d**) and liver (**e**) of HFD-AL and HFD-IF mice (n=4 mice per group). **f** H&E staining and quantification of lipid droplets of livers from

HFD-AL and HFD-IF mice (n=3 mice per group). Scale bar is 50 µm. **g, h** Western blot (**g**), measuring endogenous p53, and qPCR analysis (**h**), measuring *Trp53* and p53 target gene expression, in differentiated C3H10T1/2 cells that were kept in full growth medium (Ctrl), starved for 24 hours (STV), or starved for 24 hours and then refed for 1, 3, 6, and 24 hours (n=4 independent experiments). (**i**) Gene set enrichment analysis (GSEA) from RNA-seq data comparing p53 knock-down (siTP53) and control (siCtrl) 24 hour starved SGBS adipocytes. Dr.Tom analysis tool (BGI) was used to map GSEA hallmark gene sets (NES, normalized enrichment score). Data are presented as mean values ± SEM. Significant differences were analysed by two-tailed, unpaired t-test (**a-f**) or one-way (**h**) ANOVA for each gene with Bonferroni post hoc tests. In (**h**), * or $^{\$}$ signifies differences to the Ctrl group or the STV group, respectively. ***$^{,\ \$\$\$}P < 0.001$, **$^{,\ \$\$}P < 0.01$, and *$^{,\$}P < 0.05$. Source data are provided as a Source Data file.

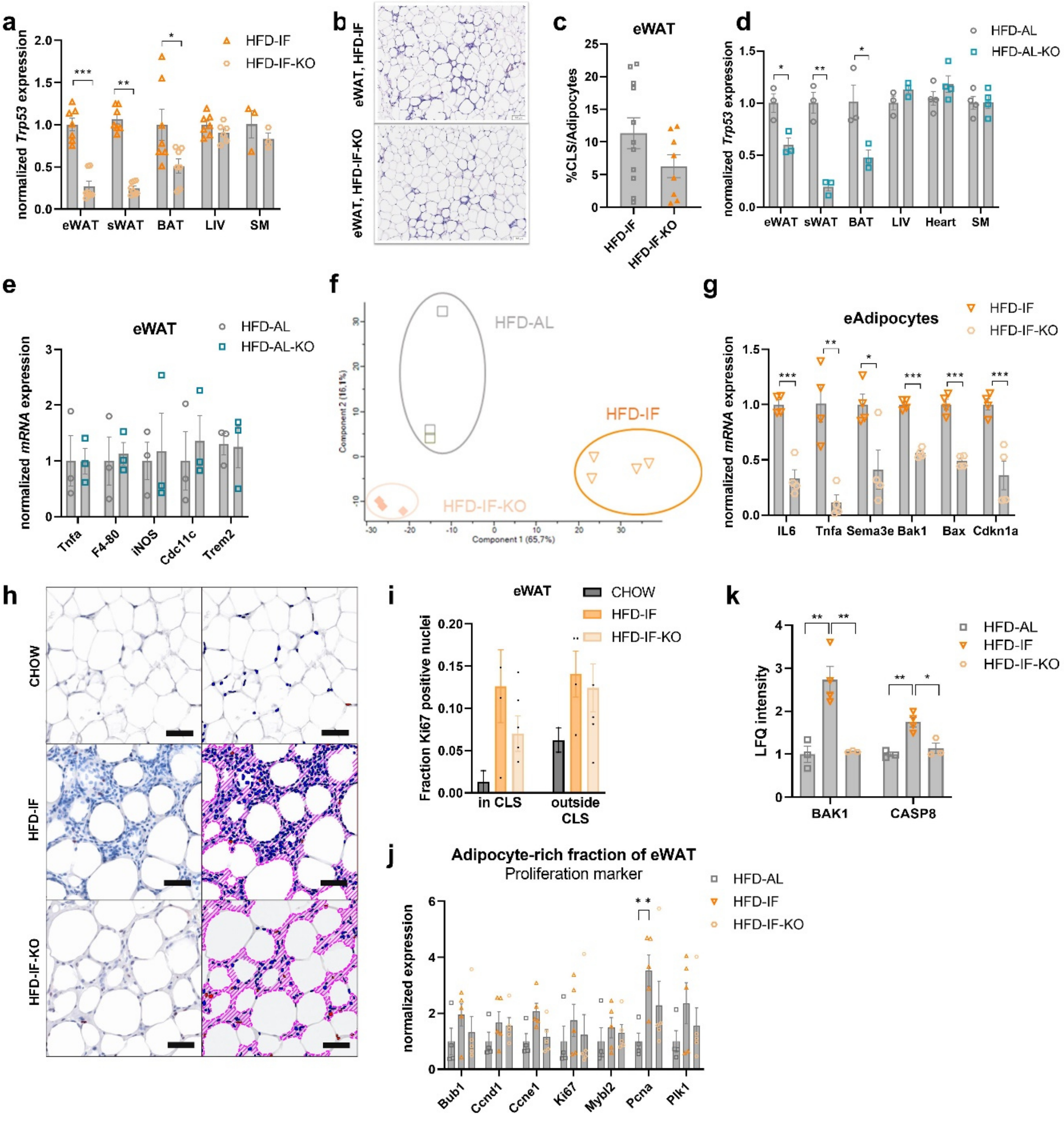

Supplementary Fig. 3 | Stress-responsive transcription factor p53 regulates eWAT remodelling in response to IF a mRNA expression of *Trp53* in eWAT (n=7 mice), sWAT (n=7 mice), BAT (n=7 mice), liver (LIV, n=7 mice) and skeletal muscle (SM, n=3 mice) of HFD-IF and HFD-IF-KO mice. **b** Representative H&E stainings in eWAT of HFD-IF and HFD-IF-KO (n=8-11 mice) mice. Scale bar is 100µm. **c** Number of crown-like structures normalized to adipocyte count in eWAT of HFD-IF (n=11 mice) and HFD-IF-KO (n=8 mice) mice. **d** mRNA expression levels of *Trp53* in eWAT (n=3 mice), sWAT (n=3 mice), BAT (n=3 mice), liver (LIV, n=4 mice), heart (n=4 mice) and skeletal muscle (SM, n=4 mice) of *ad libitum* HFD-fed Ctrl (HFD-AL) and p53 KO (HFD-AL-KO) mice. **e** mRNA expression levels of *Tnfa*, *Adgre1 (=F4-80)*, *Nos2 (=iNOS)*, *Cd11c*, and *Trem2* of HFD-AL and HFD-AL-KO mice (n=3 mice per group). **f** PCA plot of proteomic analysis showing individual mice of HFD-AL, HFD-IF and HFD-IF-KO mice. **g** mRNA expression levels of genes encoding for inflammatory or apoptotic markers in the

adipocyte-rich fraction isolated of eWAT of HFD-IF or HFD-IF-KO mice (n=4 per group). **h** Representative image magnifications of whole tissue section from mouse eWAT in HFD-IF and HFD-IF-KO mice compared to lean animals (CHOW). Immunostaining was done with anti-Ki67 (red) and counterstaining with haematoxylin (blue). Corresponding quantification results are shown in the right panels (Ki67-negative nuclei in blue, Ki67-positive nuclei in red, scale bar = 50 µm). Pink hatched areas denote identified crown-like structures (CLS). **i** Quantification of fraction of Ki67-positive nuclei within and outside of CLS (CHOW: n = 2 mice, HFD-IF: n = 4 mice, HFD-IF-KO: n = 6 mice). **j** mRNA expression levels of genes coding for proteins involved in proliferation and cell cycle in the adipocyte-rich fraction isolated from eWAT of HFD-AL (n = 4 mice), HFD-IF (n = 5 mice), or HFD-IF-KO (n = 5 mice) mice. **k** LFQ intensity for apoptotic proteins detected in the proteomics dataset in eWAT of HFD-AL (n=3 mice), HFD-IF (n=4 mice), and HFD-IF-KO (n=3 mice) mice. Data are presented as mean values ± SEM. Significant differences were analysed by two-tailed, unpaired t-test (**a, c-e, g**) or one-way ANOVA (**i-k**) with Bonferroni post hoc tests. ***P < 0.001, **P < 0.01, and *P < 0.05. Source data are provided as a Source Data file.

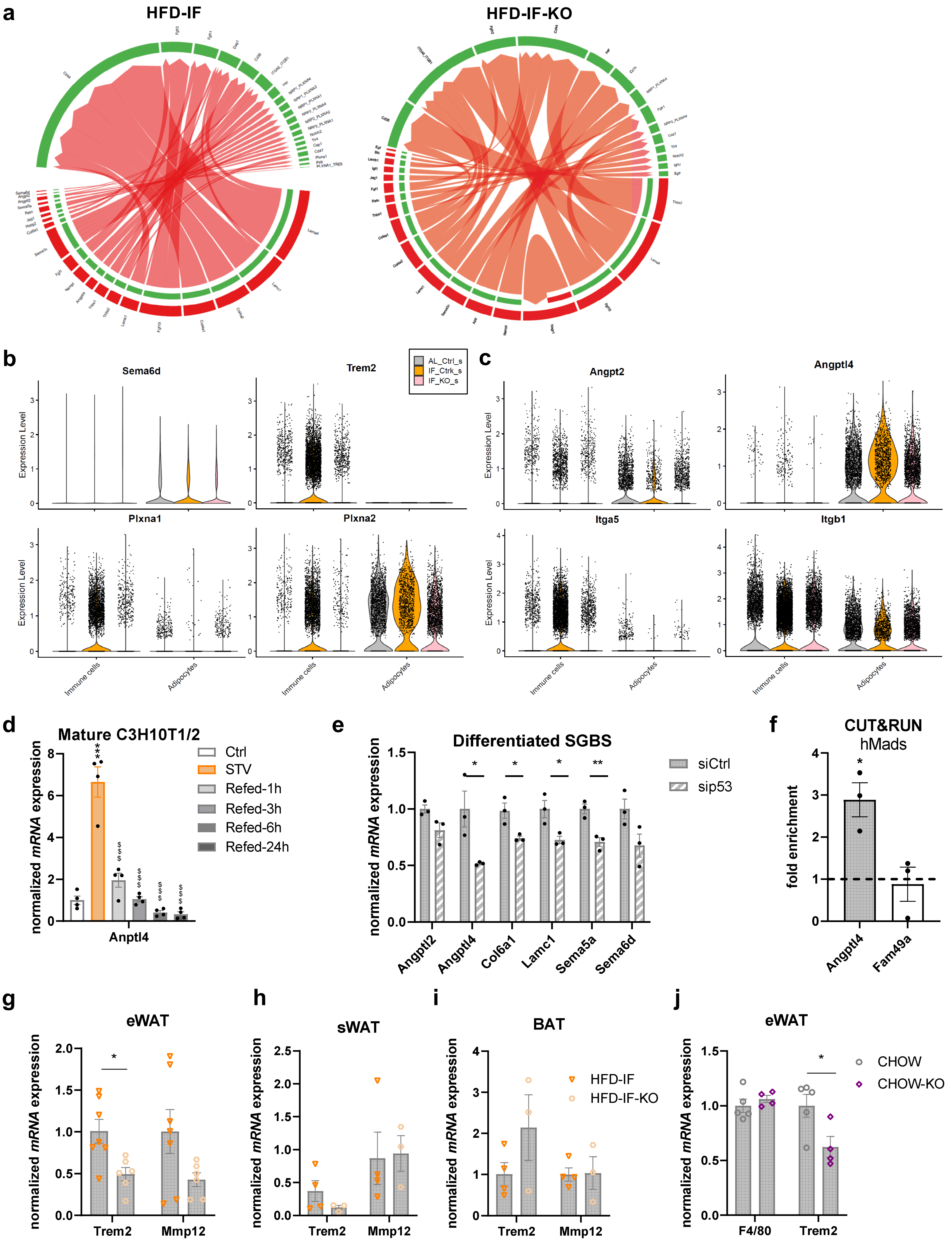

a
HFD-IF
HFD-IF-KO
b
Sema6d
Trem2
Plxna1
Plxna2
AL_Ctrl_s
IF_Ctrk_s
IF_KO_s
Expression Level
Immune cells
Adipocytes
c
Angpt2
Angptl4
Itga5
Itgb1
d
Mature C3H10T1/2
normalized mRNA expression
Ctrl
STV
Refed-1h
Refed-3h
Refed-6h
Refed-24h
Anptl4
e
Differentiated SGBS
siCtrl
sip53
Angptl2
Angptl4
Col6a1
Lamc1
Sema5a
Sema6d
f
CUT&RUN
hMads
fold enrichment
Angptl4
Fam49a
g
eWAT
Trem2
Mmp12
h
sWAT
i
BAT
HFD-IF
HFD-IF-KO
j
eWAT
F4/80
Trem2
CHOW
CHOW-KO

Supplementary Fig. 4 | Lipid-associated macrophages strikingly increase in eWAT of intermittently fasted mice. a Circle plot depicting ligand/receptor interactions between adipocytes and immune cells in eWAT HFD-IF and HFD-IF-KO mice. **b, c** Violin plots from single-nuclei RNA sequencing data showing the expression of *Sema6d*, *Trem2*, *Plxna1*, *Plxna2* (**b**), *Angptl2*, *Angptl4*, *Itga5*, *Itgb1* (**c**) in immune cells and adipocytes of eWAT of HFD-AL, HFD-IF, and HFD-IF-KO mice. **d** qPCR analysis measuring *Angptl4* gene expression in differentiated C3H10T1/2 cells that were kept in full growth medium (Ctrl), starved for 24 hours (STV), or starved for 24 hours and then refed for 1, 3, 6, and 24 hours (n = 4 independent experiments). **e** mRNA expression levels of p53 knock-down affected ligands in differentiated SGBS cells treated with siRNA targeting *Trp53* (sip53) or non-targeting siCtrl (n = 3 independent experiments). **f** Fold enrichment (target region over IgG isotype control) determined by CUT&RUN followed by qPCR targeting predicted p53 binding sides in an intronic region of *Angptl4*. Negative control primers (*Fam49a*) are designed at genomic regions that are remote from the targeted genomic region (n = 3 independent experiments). **g-i** mRNA expression levels of *Trem2* and *Mmp12* in eWAT (n=6-7 mice), sWAT (n=3-4 mice), and BAT (n=3-4 mice) of HFD-IF and HFD-IF-KO mice. **j** mRNA expression levels of *Adgre1 (=F4/80)* and *Trem2* in chow-fed, lean (Ctrl, n=5 mice) and p53 KO (n=4 mice) mice. Data are presented as mean values ± SEM. Significant differences were analysed by two-tailed, unpaired t-test (**e-j**) or one-way (**d**) ANOVA with Bonferroni post hoc tests. In (**d**), * or $^{\$}$ signifies differences to the Ctrl group or the STV group, respectively. ***$^{,\$\$\$}$P < 0.001, **P < 0.01, and *P < 0.05. Source data are provided as a Source Data file.

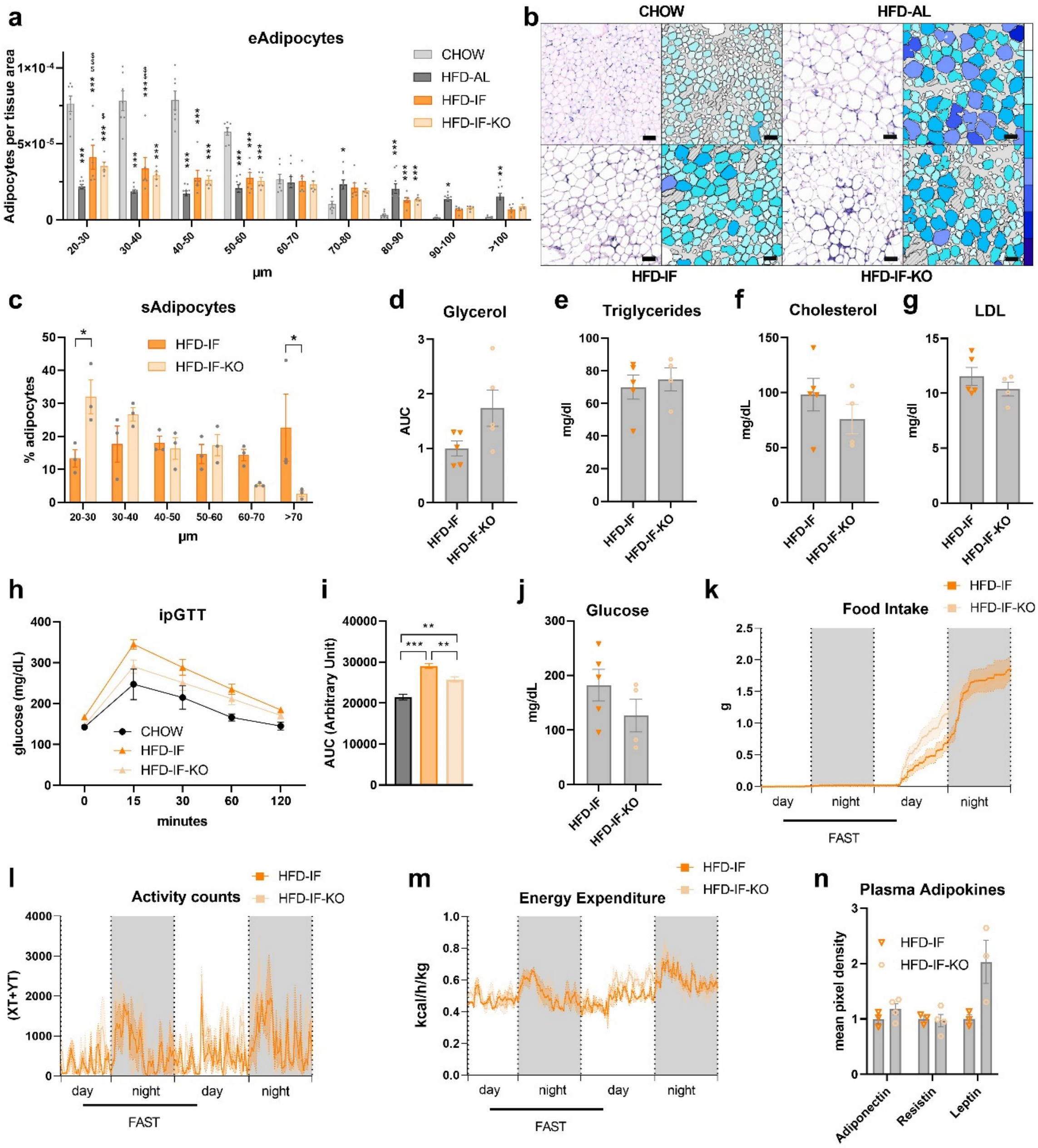

Supplementary Fig. 5 | Adipocyte p53 shapes the systemic response to IF. a Histogram showing adipocyte size distribution as quantified from whole tissue scans using a customized VisioPharm pipeline in lean (CHOW, n=7 mice), HFD-AL (n=9 mice), HFD-IF (n=6 mice), and HFD-IF-KO (n=5 mice) groups. **b** Representative images from the 4 groups with H&E staining in the left panels and quantification results showing adipocytes with different sizes in shades of blue according to the categories in (**a**) in the right panels (scale bar = 100 µm). **c** Histograms showing adipocyte diameter distributions from sWAT quantified from H&E stainings (n = 3 mice per group). **d-g** Plasma glycerol, triglycerides (TG), cholesterol (Chol) and LDL levels of HFD-IF (n=5 mice) and HFD-IF-KO (n=4 mice) mice. **h, i** Intra-peritoneal glucose tolerance test (ipGTT) of lean (n = 7 mice), HFD-IF (n = 12 mice), and HFD-IF-KO (n = 12 mice) mice. **j** Plasma glucose levels of HFD-IF (n=5 mice) and HFD-IF-KO (n=4 mice)

mice. **k-m** Food intake, activity counts, and energy expenditure of HFD-IF and HFD-IF-KO mice during a time-period of 48 h (n = 3 mice per group). **n** Plasma levels of the adipokines adiponectin, resistin, and leptin in HFD-IF (n=3 mice) and HFD-IF-KO (n=4 mice) mice. Data are presented as mean values ± SEM. Significant differences were analysed by two-tailed, unpaired t-test (**d-g, j, n**), or one-way (**i**) or two-way (**a, c, h, i, k-m**) ANOVA with Bonferroni post hoc tests. In (**a**), * or $ signifies differences to the Ctrl group or the STV group, respectively. ***, $$$P < 0.001, **, $$P < 0.01, and *,$P < 0.05. Source data are provided as a Source Data file.

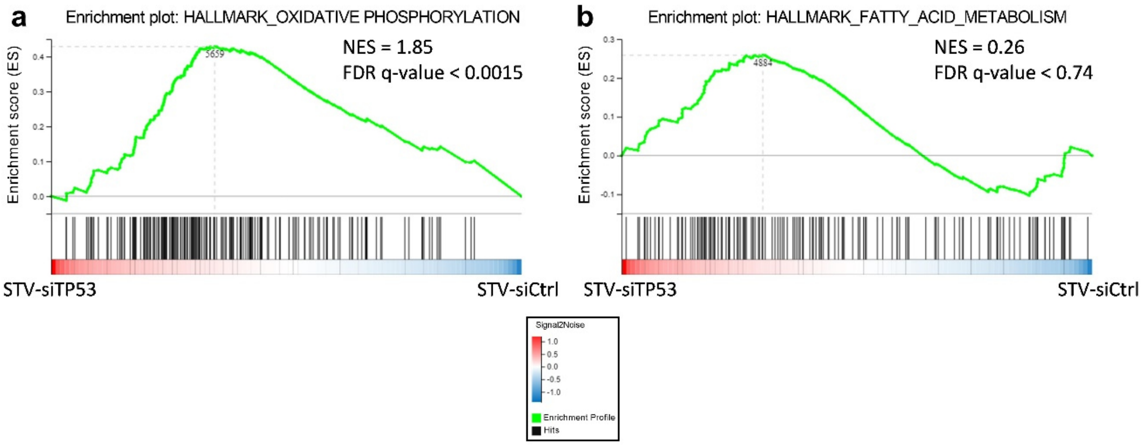

Supplementary Fig. 6 | Fatty acid oxidation and oxidative phosphorylation enrichment upon p53 knock down in starved human adipocytes. a,b Gene set enrichment analysis (GSEA) from RNA-seq data comparing p53 knock-down (siTP53) and control (siCtrl) in 24 hour starved SGBS adipocytes. Dr.Tom analysis tool (BGI) was used to map GSEA hallmark gene sets (NES, normalized enrichment score).

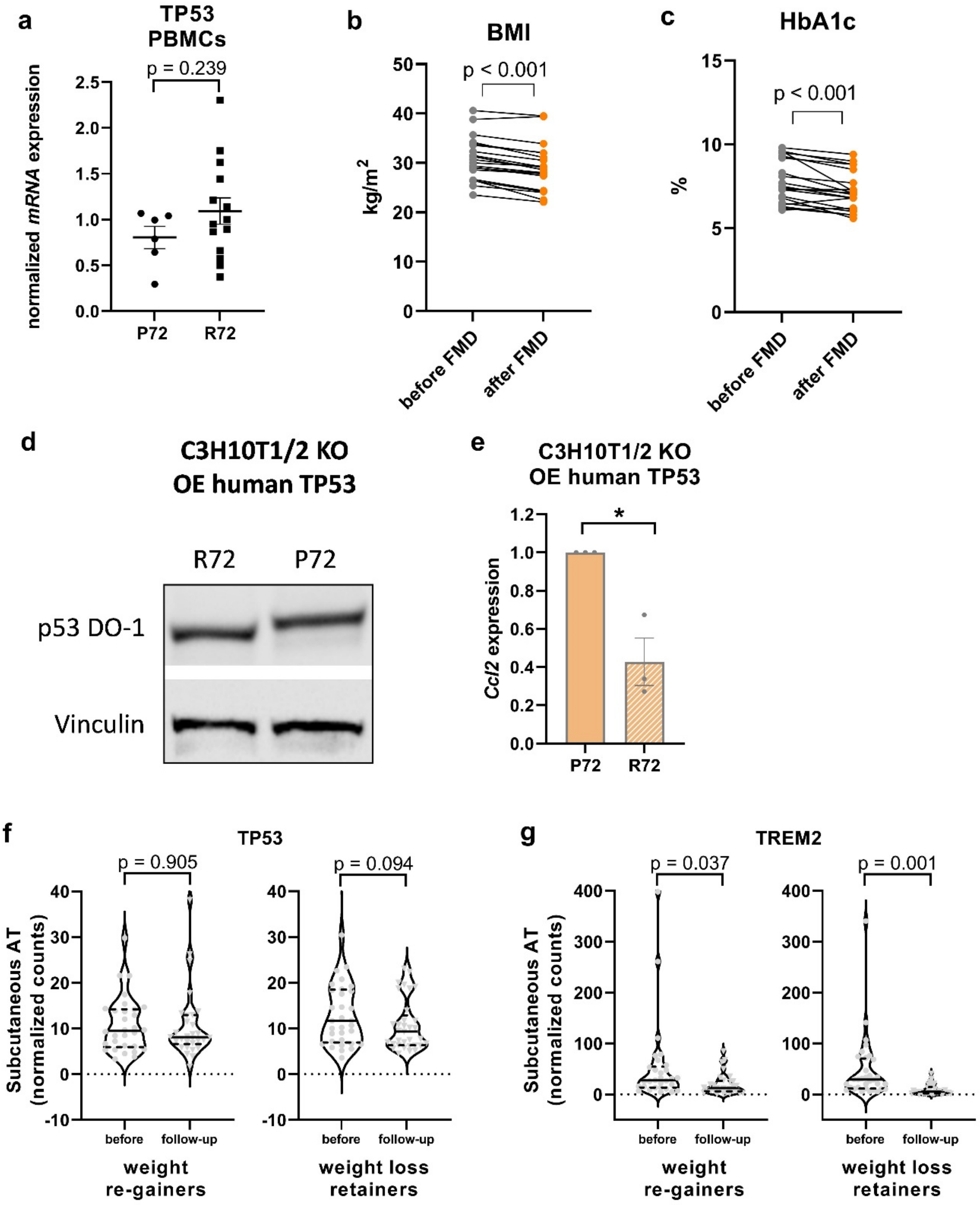

Supplementary Fig. 7 | p53 variants in diabetic patients and long-term expression after bariatric surgery. a-c BMI and plasma HbA1c levels before and after three cycles of fasting-mimicking diet (FMD) in a cohort of diabetic patients stratified by P72 (n=6 patients) and R72 (n=14 patients) TP53 polymorphism. **d, e** Differentiated mouse C3H10T1/2 adipocytes with CRISPR-mediated p53 KO were used to overexpress human TP53 harbouring either the R72 or the P72 variant using electroporation. Twenty-four hours after electroporation cells were kept in starvation medium (HBSS+HEPES) for

another 24 hours (n=3 per group, from three independent experiments). (**d**) shows equal overexpression from the 2 plasmids. (**e**) shows significantly reduced *Ccl2* mRNA expression in samples overexpressing the R72 variant. **f, g** RNA-seq expression of *TP53* and the LAM marker *TREM2* in visceral and subcutaneous AT from obese subjects before they underwent bariatric surgery and after follow-up of two years. Comparison between weight re-gainers (BMI<25% loss retained, n=30 patients) and weight loss retainers (BMI>=25% loss retained, n=33 patients). Data are presented as mean values ± SEM. Significant differences were analysed by two-tailed, unpaired (**a**) or paired t-test (**b-c, f, g**) or one-sample t-test (**e**). *P < 0.05. Source data are provided as a Source Data file.

Supplemenatary Table 1 (oligonucleotide sequences)

Oligonucleotides	Source	fwd (5'-3')	rev (5'-3')
mTnfa	Microsynth	GGCAGGTCTACTTTGGAGTCATTGC	ACATTCGAGGCTCCAGTGAATTCGG
mIl6	Microsynth	GACAACCACGGCCTTCCCTA	GCCTCCGACTTGTGAAGTGGT
mNos2	Microsynth	GGCAAACCCAAGGTCTACGTT	TCGCTCAAGTTCAGCTTGGT
mCcl2	Microsynth	TTAAAAACCTGGATCGGAACCAA	GCATTAGCTTCAGATTTACGGGT
mCd11c	Microsynth	GGAGGAGAACAGAGGTGCTG	GGAGGAGAACAGAGGTGCTG
mMmp12	Microsynth	GGGCTGCTCCCATGAATGAC	CCAGAGTTGAGTTGTCCAGTTG
mTrem2	Microsynth	CTGGAACCGTCACCATCACTC	CGAAACTCGATGACTCCTCGG
mLipa	Microsynth	TGTTCGTTTTCACCATTGGGA	CGCATGATTATCTCGGTCACA
mGpnmb	Microsynth	GCTGGTCTTCGGATGAAAATGA	CCACAAAGGTGATATTGGAACCC
mLgals3	Microsynth	AGACAGCTTTTCGCTTAACGA	GGGTAGGCACTAGGAGGAGC
mBak1	Microsynth	TGCCTACGAACTCTTCACCAA	TGGTAGACGTACAGGGCCAG
mBax	Microsynth	TAGCAAACTGGTGCTCAAGG	TCTTGGATCCAGACAAGCAG
mBcl2	Microsynth	GCTACCGTCGTGACTTCGC	CCCCACCGAACTCAAAGAAGG
mPuma	Microsynth	GTACGAGCGGCGGAGACAAG	GCACCTAGTTGGGCTCCATTTCTG
mp16	Microsynth	CAGATTCGAACTGCGAGGA	CAGCGGAACACAAAGAGCA
mParp1	Microsynth	TGGTTTCAAGTCCCTTGTCC	TGCTGTCTATGGAGCTGTGG
mTrp53	Microsynth	ACATGACGGAGGTCGTGAG	AATTTCCTTCCACCCGGATA
mCdkn1a	Microsynth	CCTGGTGATGTCCGACCTG	CCATGAGCGCATCGCAATC
mMdm2	Microsynth	AGCAGCGAGTCCACAGAGAC	ATCCTGATCCAGGCAATCAC
mMgl	Microsynth	GCAGCCACTAGGATGGAGATG	GCAATGTAGAACCCCAGAACAC
mHsl	Microsynth	CCATCTCACCTCCCTTGG	TCCTTCCCGTAGGTCATAGG
mAtgl	Microsynth	GAGACCAAGTGGAACATC	GTAGATGTGAGTGGCGTT
mCgi58	Microsynth	GGTTAAGTCTAGTGCAGC	AAGCTGTCTCACCACTTG
mAdrb3	Microsynth	GGTAGTGGGACTCCTCGTAATG	GGGTTGGTGACAGCTAGGTA
mF4/80	Microsynth	TGGCTGCCTCCCTGACTTTC	CAAGATCCCTGCCCTGCACT
mSema3a	Microsynth	ATCAGTGGGTGCCTTACCAA	GCCAAATGTTTTACTGGGACA
hTP53	Microsynth	CAGCACATGACGGAGGTTGT	TCATCCAAATACTCCACACGC
hMdm2	Microsynth	GAATCATCGGACTCAGGTACATC	TCTGTCTCACTAATTGCTCTCCT
hCdkn1a	Microsynth	GGCAGACCAGCATGACAGATT	GCGGATTAGGGCTTCCTCTT
hBak	Microsynth	ATGGTCACCTTACCTCTGCAA	TCATAGCGTCGGTTGATGTCG
hBcl2	Microsynth	CGATGCCTTTGTGGAACTGT	AGCCTGCAGCTTTGTTTCAT
hBax	Microsynth	TTTTGCTTCAGGGTTTCATC	CAGTTGAAGTTGCCGTCAGA
hAngptl2	Microsynth	TCCTGCACGAGATCATCCG	GGTGCTGGTACTTGTGCTCC
hAngptl4	Microsynth	GGCTCAGTGGACTTCAACCG	CCGTGATGCTATGCACCTTCT
hCol6a1	Microsynth	ACAGTGACGAGGTGGAGATCA	GATAGCGCAGTCGGTGTAGG
hLamc1	Microsynth	GGACTCCGCCCGAGGAATA	ACTTGAGACGCACATAGGTGA
hSema5a	Microsynth	GGAACCTGTGTTATAGCATGGC	GCACTGAGTCGTACCCTGG
hSema6d	Microsynth	AGGCAATATCCGGTTTTTAGAGG	CCCTGCCAGCAATATAAAGTGT
mPlk1	Microsynth	CCCGCTGGCGAAAGAAATTC	CATTTGGCGAAGCCTCCTTTA
mMybl2	Microsynth	TCTGGATGAGTTACACTACCAGG	GTGCGGTTAGGAAAGTGACTG
mBub1	Microsynth	AGAATGCTCTGTCAGCTCATCT	TGTCTTCACTAACCCACTGCT
mCcne1	Microsynth	GTGGCTCCGACCTTTCAGTC	CACAGTCTTGTCAATCTTGGCA
mCcnd1	Microsynth	GCGTACCCTGACACCAATCTC	CTCCTCTTCGCACTTCTGCTC
mKi67	Microsynth	ATCATTGACCGCTCCTTTAGGT	GCTCGCCTTGATGGTTCCT
mPcna	Microsynth	TTTGAGGCACGCCTGATCC	GGAGACGTGAGACGAGTCCAT
mMdm2	Microsynth	ATCCACCACCGCTTCAAGAAC	TACCACCAAACTCTCACGGTT
hAngptl4_cut	Microsynth	GGC CTC TCC GTA CCC TTC TC	AGA TAA GGA GGG AAG CTG CTG G
hFam49a_cut	Microsynth	GCA AAG AGA TTC CAG CAT CC	CAT TTG CAT CTG CAT CCT GT
