# Supplementary material for: Adipocyte p53 coordinates the response to intermittent fasting by regulating adipose tissue immune cell landscape
Source: Nat Commun. 2024 Feb 15;15:1391. doi: 10.1038/s41467-024-45724-y (PMC10869344; doi:10.1038/s41467-024-45724-y)
Supplement: Supplementary file 3 — Description of Additional Supplementary Files [file 41467_2024_45724_MOESM3_ESM.pdf]

Description of Additional Supplementary Files

Title: Supplementary Data 1

Description: Differentially expressed genes between HFD-AL and HFD-IF group within the major cell types derived from the sn-RNAseq data set.

Title: Supplementary Data 2

Description: Differentially expressed genes between HFD-IF and HFD-IF-KO group within the major cell types derived from the sn-RNAseq data set.
