# Supplementary material for: Adipocyte p53 coordinates the response to intermittent fasting by regulating adipose tissue immune cell landscape
Source: Nat Commun. 2024 Feb 15;15:1391. doi: 10.1038/s41467-024-45724-y (PMC10869344; doi:10.1038/s41467-024-45724-y)
Supplement: Supplementary file 6 — Reporting Summary [file 41467_2024_45724_MOESM6_ESM.pdf]

nature portfolio

Corresponding author(s): Andreas Prokesch

Last updated by author(s): Jan 25, 2024

Reporting Summary

Nature Portfolio wishes to improve the reproducibility of the work that we publish. This form provides structure for consistency and transparency in reporting. For further information on Nature Portfolio policies, see our Editorial Policies and the Editorial Policy Checklist.

Statistics

For all statistical analyses, confirm that the following items are present in the figure legend, table legend, main text, or Methods section.

n/a	Confirmed	
[ ]	[x]	The exact sample size (n) for each experimental group/condition, given as a discrete number and unit of measurement
[ ]	[x]	A statement on whether measurements were taken from distinct samples or whether the same sample was measured repeatedly
[ ]	[x]	The statistical test(s) used AND whether they are one- or two-sided<br>*Only common tests should be described solely by name; describe more complex techniques in the Methods section.*
[x]	[ ]	A description of all covariates tested
[ ]	[x]	A description of any assumptions or corrections, such as tests of normality and adjustment for multiple comparisons
[ ]	[x]	A full description of the statistical parameters including central tendency (e.g. means) or other basic estimates (e.g. regression coefficient) AND variation (e.g. standard deviation) or associated estimates of uncertainty (e.g. confidence intervals)
[ ]	[x]	For null hypothesis testing, the test statistic (e.g. F, t, r) with confidence intervals, effect sizes, degrees of freedom and P value noted<br>*Give P values as exact values whenever suitable.*
[x]	[ ]	For Bayesian analysis, information on the choice of priors and Markov chain Monte Carlo settings
[x]	[ ]	For hierarchical and complex designs, identification of the appropriate level for tests and full reporting of outcomes
[x]	[ ]	Estimates of effect sizes (e.g. Cohen's d, Pearson's r), indicating how they were calculated

Our web collection on statistics for biologists contains articles on many of the points above.

Software and code

Policy information about availability of computer code

Data collection	No custom algorithm or software were used for data collection.
Data analysis	No custom algorithm or software were used for data analysis.<br>Other algorithms and softwares used in this study:<br>Prism 9.0, GraphPad<br>Perseus v1.6.14, Perseus (maxquant.net)<br>Seurat, https://satijalab.org/seurat/<br>Cellbender, cellbender.rtfd.io<br>Maxquant v1.6.17 , https://maxquant.net/maxquant/<br>EnrichR, Enrichr (maayanlab.cloud)<br>Doubletfinder (https://satijalab.org/seurat/)<br>Kallisto v0.46, pachterlab.github.io/kallisto<br>ImageJ, https://imagej.net/software/imagej/<br>VisioPharm version 2019.09, https://visiopharm.com/<br>DEseq2 (v1.34.1), bioconductor.org/packages/DESeq2<br>R (v4.0.2), RRID: SCR_001905

For manuscripts utilizing custom algorithms or software that are central to the research but not yet described in published literature, software must be made available to editors and reviewers. We strongly encourage code deposition in a community repository (e.g. GitHub). See the Nature Portfolio guidelines for submitting code & software for further information.

Data

Policy information about availability of data

All manuscripts must include a data availability statement. This statement should provide the following information, where applicable:

- Accession codes, unique identifiers, or web links for publicly available datasets
- A description of any restrictions on data availability
- For clinical datasets or third party data, please ensure that the statement adheres to our policy

The sequencing data generated in this study have been deposited in the Gene Expression Omnibus (GEO) repository under accession code GSE229690 (https://www.ncbi.nlm.nih.gov/geo/query/acc.cgi?acc=GSE229690) for the snRNA-Seq dataset and GSE248893 (https://www.ncbi.nlm.nih.gov/geo/query/acc.cgi?acc=GSE248893) for the RNA-sequencing dataset of SGBS adipocytes. The proteomic data was deposited at PRIDE repository under the accession number PXD041351 (https://www.ebi.ac.uk/pride/archive/projects/PXD041351). Any additional information required to reanalyse the data reported in this paper is available from the lead contact upon request. Source data are provided with this paper.

Research involving human participants, their data, or biological material

Policy information about studies with human participants or human data. See also policy information about sex, gender (identity/presentation), and sexual orientation and race, ethnicity and racism.

Reporting on sex and gender	Data from human cohorts were analysed retrospectivly. Details on the FMD cohort were published previously (DOI: 10.1210/clinem/dgac197) and registered under German Clinical Trials Register (Deutsches Register Klinischer Studien DRKS), DRKS-ID: DRKS00014287), and included 6 females and 14 males (age 52-75) who signed informed consents. For the bariatric surgery cohort, biometric data as well as visceral and subcutaneous AT samples from the Leipzig Obesity Biobank were kindly provided by Matthias Blüher (Department of Medicine, University of Leipzig). AT biopsies were obtained from 135 individuals (37 men, 98 women, age ??) with morbid obesity in the context of a two-step bariatric surgery approach.
Reporting on race, ethnicity, or other socially relevant groupings	Recruitment was done via German hospitals. No selection bias was applied.
Population characteristics	FMD cohort: German Clinical Trials Register (Deutsches Register Klinischer Studien DRKS), DRKS-ID: DRKS00014287
Recruitment	Patients were recruited if diagnosed with type 2 diabetes mellitus (FMD cohort) or with morbid obesity qualifying the for bariatric surgery.
Ethics oversight	FMD cohort: University Hospital of Heidelberg (Ethic-Nr. S-682/2016)<br>Bariatric surgery cohort: Ethics Committee of the University of Leipzig (approval number 159-12-21052012). The study was performed in agreement with the Declaration of Helsinki.<br>All study participants gave written consent to use their data in an anonymized form for research purposes before taking part in this study.

Note that full information on the approval of the study protocol must also be provided in the manuscript.

Field-specific reporting

Please select the one below that is the best fit for your research. If you are not sure, read the appropriate sections before making your selection.

- [x] Life sciences
- [ ] Behavioural & social sciences
- [ ] Ecological, evolutionary & environmental sciences

For a reference copy of the document with all sections, see nature.com/documents/nr-reporting-summary-flat.pdf

Life sciences study design

All studies must disclose on these points even when the disclosure is negative.

Sample size	The authors declare that no statistical models were used to determine sample size. Nearly all in vivo studies included a sample size of at least five mice per group, which was typically sufficient to determine statistical significance between groups, according to our prior experience and community standards. All data from in vitro experiments shown were performed in at least three independent experiments.
Data exclusions	Significant outliers were excluded if they were identified by the "extreme studentized deviate" method.
Replication	All experiments were replicated in at least three individual independent experiments or subjects. Most results were confirmed with complementary methods (e.g. snRNA-seq and protoemics and qPCR).
Randomization	All groups were assigned randomly.
Blinding	Blinding was performed for quantification of histological images. All other data needed to be unblinded upon data analysis for group assignment.

Reporting for specific materials, systems and methods

We require information from authors about some types of materials, experimental systems and methods used in many studies. Here, indicate whether each material, system or method listed is relevant to your study. If you are not sure if a list item applies to your research, read the appropriate section before selecting a response.

Materials & experimental systems

n/a	Involved in the study
☐	☒ Antibodies
☐	☒ Eukaryotic cell lines
☒	☐ Palaeontology and archaeology
☐	☒ Animals and other organisms
☐	☒ Clinical data
☒	☐ Dual use research of concern
☒	☐ Plants

Methods

n/a	Involved in the study
☒	☐ ChIP-seq
☒	☐ Flow cytometry
☒	☐ MRI-based neuroimaging

Antibodies

Antibodies used

p53 (32532 (D2H9O), 1:1000 Cell Signalling, Danvers, MA, United States)
p53 (sc-126 (DO-1), 1:3000, Santa Cruz Biotechnology, Dallas, Texas, United States)
GAPDH (2118 (14C10), 1:5000, Cell Signalling, Danvers, MA, United States)
β-actin (Ab6276 (AC-15), 1:250000, Abcam, Cambridge, United Kingdom)
vinculin (PA5-29688, 1:1000, Thermo Fisher Scientific, Waltham, MA, United States)
MMP12 (ab52897 (EP1261Y), 1:1000, Abcam, Cambridge, United Kingdom)
cleaved caspase 3 antibody (9661, 1:50, Cell Signalling Technology, Denver, MA, USA)
Ki67 antibody (12202, 1:400, Cell Signalling Technology, Denver, MA, USA)
Goat anti-Rabbit IgG (31460, 1:5000, Thermo Fisher Scientific, Waltham, MA, United States) and Goat anti-Mouse IgG (31430, 1:5000, Thermo Fisher Scientific, Waltham, MA, United States) were used as secondary HRP-conjugated antibodies.

Validation

p53 antibodies were validated with knock out in cell lines (C3H10T1/2 for mouse (see source data) and HepG2 for human antibody). MMP12 antibody was validated in knock-out mice (Melina Amor, Dagmar Kratky). Immunohistochemistry and CUT&RUN was validated with IgG negative conrols.

Eukaryotic cell lines

Policy information about cell lines and Sex and Gender in Research

Cell line source(s)

The cell lines were authenticated by the providers. Only low-passage cells were used for experiments.
C3H10T1/2 clone 8 mouse mesenchymal stem cells were purchased from ATCC (CCL-226, ATCC, Virginia, United States).
Human Simpson-Golabi-Behmel syndrome (SGBS) preadipocyte cells were kindly provided by Martin Wabitsch.
Human multipotent adipose-derived stem cells (hMADs) were cultivated and differentiated to white adipocytes as previously described [Elabd, C. et al. Human multipotent adipose-derived stem cells differentiate into functional brown adipocytes. Stem Cells 27, 2753–2760 (2009).].
For stromal vascular fraction (SVF) isolation sWAT depots from male mice were harvested.

Authentication

None of the used cell lines are to be found in the ICLAC list and therefore not authenticated by us, but by the vendors.

Mycoplasma contamination

All cell lines were tested mycoplasm negative using Venor GeM Classic (Minerva Biolabs).

Commonly misidentified lines
(See ICLAC register)

None of the used cell lines are to be found in the ICLAC list.

Animals and other research organisms

Policy information about studies involving animals; ARRIVE guidelines recommended for reporting animal research, and Sex and Gender in Research

Laboratory animals

Mouse: Adiponectin-CreERT2 (C57Bl/6J background), gift from Tim J. Schulz via MTA from Pierre Chambon
Mouse: p53-Lox , National Cancer Institute (NCI) Mouse Repository, FVB.129P2- Trp53tm1Brn/Nci; Stock No. 01XC2
Mouse: C57Bl/6J, In house breeding
Breeding was done with mice older than 10 weeks.
Experiments were started by feeding a 60% HFD in 5 weeks old mice. Mice were sacrificed at the age of 31 weeks at the latest.

Wild animals	No wild animals were studied.
Reporting on sex	Only male mice were used in this study as female mice gain less weight on a high-fat diet and were less responsive to tamoxifen-induced p53 ablation, which was validated in preliminary studies.
Field-collected samples	No field collected samples were used in the study.
Ethics oversight	All animal studies were approved by the Austrian Ministry for Education, Science and Research (Vienna, Austria, BMWFW-66.010/0087-WF/V/3b/2017) and performed strictly according to its guidelines.

Note that full information on the approval of the study protocol must also be provided in the manuscript.

Clinical data

Policy information about clinical studies
All manuscripts should comply with the ICMJEguidelines for publication of clinical research and a completedCONSORT checklist must be included with all submissions.

Clinical trial registration	No clinical trial was instigated for this work. Data was analysed retrospectively.
Study protocol	*Note where the full trial protocol can be accessed OR if not available, explain why.*
Data collection	*Describe the settings and locales of data collection, noting the time periods of recruitment and data collection.*
Outcomes	*Describe how you pre-defined primary and secondary outcome measures and how you assessed these measures.*
